# Predictors of Cancer Genetic Testing Among U.S. Adults: Insights From the Health Information National Trends Survey 7

**DOI:** 10.7759/cureus.95699

**Published:** 2025-10-29

**Authors:** Ananya Roy, Preksha Singh, Rushi Vaghela, Naqeeya Sabuwala, Abhi Shah, Shreyans Singhvi, Prudence Lam

**Affiliations:** 1 Internal Medicine, Smt. Nathiba Hargovandas Lakhmichand Municipal Medical College, Ahmedabad, IND; 2 Internal Medicine, G.B. Clinic, Jodhpur, IND; 3 Oncology, Mount Auburn Hospital, Harvard Medical School, Cambridge, USA

**Keywords:** cancer, genetic testing, genomics, health disparities, health information national trends survey (hints), hereditary cancer, predictors, screening uptake, sociodemographic factors

## Abstract

Background: Progress in molecular genomics has increased the accessibility, availability, and affordability of genetic testing to correctly identify individuals at elevated risk for hereditary cancers. Understanding the sociodemographic, clinical, and behavioural factors influencing genetic testing uptake is essential to addressing disparities and guiding targeted interventions.

Aim and objective: This study aimed to assess the prevalence of cancer genetic testing in U.S. adults, while correlating it with various predictors of clinical and behavioural aspects, using data from the 2024 Health Information National Trends Survey 7 (HINTS 7).

Methods: We analysed data from the HINTS 7, a nationally representative, cross-sectional survey conducted in 2024. The analytic cohort included 514 adult respondents, corresponding to an estimated 20.7 million U.S. adults. Survey-weighted bivariate analyses were used to examine patterns of genetic testing. Additionally, multivariable logistic regression estimated adjusted odds ratios (aORs) for associations between predictors and the likelihood of undergoing cancer genetic testing using Stata 19 (StataCorp LLC, College Station, TX).

Results: Overall, 23.4% of U.S. adults reported having undergone genetic testing. Compared to respondents aged 18-34, those aged 35-49 (aOR = 4.69, 95% CI: 1.90-11.56) and 50-64 (aOR = 6.60, 95% CI: 2.67-16.33) were significantly more likely to have been tested. Non-Hispanic Black adults (aOR = 5.55, 95% CI: 2.31-13.35) and individuals of “Other” racial/ethnic backgrounds (aOR = 4.37, 95% CI: 1.46-13.06) also had higher odds compared to non-Hispanic Whites. Participants with annual household incomes between $20,000 and $34,999 (aOR = 3.80, 95% CI: 1.43-10.11) and those with a personal history of cancer (aOR = 4.32, 95% CI: 1.86-9.99) were more likely to have undergone testing. Notably, higher trust in healthcare providers was inversely associated with testing (aOR = 0.18, 95% CI: 0.04-0.87), whereas use of health/wellness apps increased the odds (aOR = 2.12, 95% CI: 1.05-4.26).

Conclusions: Age, race/ethnicity, income, personal cancer history, trust in healthcare providers, and engagement with digital health tools were significant predictors of cancer genetic testing. These findings underscore the need for targeted outreach and education, particularly among younger adults, certain racial/ethnic groups, and individuals with lower engagement in digital health, to ensure equitable access to genetic testing.

## Introduction

Over the past few decades, rapid advancements in molecular genomics have transformed healthcare by improving the accuracy, affordability, and accessibility of genetic testing [1]. These developments have enabled the early identification of individuals at increased risk for inherited disorders, opening opportunities for preventive strategies and personalised care. In oncology, genetic testing has become an indispensable tool for recognising high-risk individuals who may benefit from targeted surveillance, preventive interventions, and precision therapies [2,3].

The clinical value of cancer genetic testing is well recognised. The U.S. Centres for Disease Control and Prevention’s Office of Public Health Genomics (OPHG) has designated testing for hereditary breast and ovarian cancer (HBOC) syndrome and Lynch syndrome as Tier 1 genomic applications [4,5]. This designation reflects strong evidence supporting their use in routine clinical practice, with proven effectiveness in reducing cancer-related morbidity and mortality through early detection and timely intervention.

The benefits of genetic testing extend beyond the individual tested. A positive result can identify at-risk biological relatives who may require preventive care, enhanced surveillance, or early therapeutic interventions [6]. This “cascade testing” approach amplifies the public health benefits of genomic screening, improving outcomes across entire families.

Despite increasing availability and integration into oncology practice, utilisation of genetic testing remains uneven. Factors such as socioeconomic status, education level, health literacy, and healthcare access significantly influence testing uptake [7,8]. Cancer risk education has been incorporated into multiple health profession curricula, underscoring the importance of providing accurate, culturally sensitive, and accessible risk information to all individuals, regardless of their immediate eligibility or personal interest in testing [9]. Nonetheless, disparities persist, particularly among racial and ethnic minorities, rural communities, and populations with limited economic resources [9,10].

Identifying the sociodemographic, clinical, and behavioural factors associated with cancer genetic testing uptake is essential for addressing these inequities. A nuanced understanding of these predictors can inform targeted outreach, guide healthcare policy, and support equitable integration of genetic testing into routine cancer prevention and care. In this context, the present study uses nationally representative data from the Health Information National Trends Survey 7 (HINTS 7) to examine the prevalence and predictors of cancer genetic testing among U.S. adults, with the goal of highlighting underserved populations and identifying potential barriers to access.

## Materials and methods

We analysed public-use data from the Health Information National Trends Survey 7 (HINTS 7, 2024), a nationally representative, cross-sectional survey of non-institutionalised U.S. adults administered by the National Cancer Institute. HINTS 7 employed a stratified, address-based sampling frame and standard multi-contact mail protocols. Data for the cycle analysed were collected throughout 2024. The dataset includes design variables (strata and primary sampling units) and person-level weights that support design-correct, nationally representative estimation.

The analytic cohort comprised respondents with non-missing data on the outcome (ever having had a genetic test to assess disease risk) and all covariates included in the multivariable model. After exclusions for missingness on the dependent variable and key predictors, the final unweighted sample included 514 adults, representing an estimated 20.7 million U.S. adults when survey weights were applied. “Don’t know/refused” responses were treated as missing, and we used complete-case analysis (listwise deletion) for multivariable modelling. Because some subgroups were small, estimates for these categories were interpreted with attention to the width of confidence intervals rather than statistical significance alone.

The primary outcome was a self-reported history of cancer genetic testing (yes/no), based on the HINTS item asking whether the respondent had ever had a genetic test to assess risk for disease. Predictors were selected a priori from prior literature and conceptual relevance. Sociodemographic variables included age (18-34, 35-49, 50-64, ≥65 years), sex (male, female), race/ethnicity (non-Hispanic White, non-Hispanic Black, Hispanic, non-Hispanic Asian, Other), and household income (≤$10,000; $10,000-$19,999; $20,000-$34,999; $35,000-$74,999; ≥$75,000). Clinical and behavioural correlates included personal history of cancer, family history of cancer, self-rated health (good/excellent vs fair/poor), trust in healthcare providers (“extremely confident” vs all other responses), use of health/wellness apps (yes/no), having looked for health information online (yes/no), and difficulty paying medical bills (yes/no). Categorisations followed HINTS public-use coding; for interpretation, categories were chosen to reflect common cut-points in the genetic testing and cancer prevention literature and to maintain sufficient cell sizes.

All analyses were conducted in Stata 19 (StataCorp LLC, College Station, TX) using survey procedures to account for the complex design. We specified the person-level weight, strata, and primary sampling unit identifiers and used Taylor series linearisation for variance estimation. Weighted descriptive statistics (proportions with 95% confidence intervals) characterised the sample. Design-corrected bivariate associations with the outcome were evaluated using Wald/F tests. We then fit a survey-weighted multivariable logistic regression model with all covariates entered simultaneously to obtain adjusted odds ratios (aORs) and 95% confidence intervals for the association between each predictor and ever undergoing cancer genetic testing. The model included main effects only; we did not estimate interaction terms, perform formal model diagnostics, or undertake sensitivity analyses. All hypothesis tests were two-sided with α = 0.05, and we report exact p-values alongside aORs and 95% confidence intervals. No adjustment for multiple comparisons was made, given the exploratory, hypothesis-generating aims of the study.

The HINTS protocol was reviewed and approved by the Westat Institutional Review Board, and data collection adhered to human-subjects protections, including informed consent and respondent privacy safeguards. Because HINTS data are de-identified and publicly available, this secondary analysis was exempt from additional IRB review. Reporting follows STROBE recommendations for cross-sectional studies.

## Results

A total of 514 respondents were included in the analysis, representing an estimated 20.7 million U.S. adults. Overall, 23.4% reported having undergone genetic testing for cancer risk.

In regard to non-modifiable characteristics (Table 1), age was a significant predictor of genetic testing uptake. Compared with adults aged 18-34 years, those aged 35-49 years had more than four times higher odds of testing (aOR = 4.69; 95% CI: 1.90-11.56; p = 0.001), and those aged 50-64 years had more than six times higher odds (aOR = 6.60; 95% CI: 2.67-16.33; p < 0.001).

**Table 1:** Significance of non-modifiable characteristics linked to the incidence of individuals undergoing genetic testing for cancer. aOR: adjusted odds ratio from survey-weighted multivariable logistic regression; CI: confidence interval. Reference groups shown in parentheses. Significance defined as p < 0.05.

Predictor	aOR	95% CI	p-value
Age 18-35 (ref)	1.00 (ref)		
Age 35-49	4.69	1.90-11.56	0.001
Age 50-64	6.6	2.67-16.33	<0.001
Age≥65	3.63	0.86-15.33	0.08
Male (ref)	1.00 (ref)		
Female	1.44	0.73-2.85	0.296
Non-Hispanic White (ref)	1.00 (ref)		
Non-Hispanic Black	5.55	2.31-13.35	<0.001
Hispanic	2.26	0.94-5.40	0.068
Non-Hispanic Asian	5.07	0.75-34.13	0.095
Race ethnicity Other	4.37	1.46-13.06	0.008
Personal history of cancer	4.32	1.86-9.99	0.001
Family history of cancer	0.82	0.43-1.59	0.56
Self-rated health	0.75	0.38-1.49	0.408

Respondents aged ≥65 years had higher odds than the reference group, but this was not statistically significant (aOR = 3.63; 95% CI: 0.86-15.33; p = 0.080). Sex was not significantly associated with genetic testing. In contrast, race/ethnicity showed notable differences. Compared to non-Hispanic White adults, non-Hispanic Black respondents had more than five times higher odds of undergoing testing (aOR = 5.55; 95% CI: 2.31-13.35; p < 0.001), and those identifying as “Other” racial/ethnic background had over four times higher odds (aOR = 4.37; 95% CI: 1.46-13.06; p = 0.008).

Odds were elevated but not statistically significant for Hispanic and non-Hispanic Asian participants. A personal history of cancer was strongly associated with higher testing uptake (aOR = 4.32; 95% CI: 1.86-9.99; p = 0.001).

Family history of cancer, self-rated health status, difficulty paying medical bills, and seeking health information online were not significantly associated.

In regard to modifiable factors (Table 2), Household income was significantly associated with testing only in the USD $20,000-$34,999 category, which had 3.8 times higher odds compared with those earning <$10,000 (aOR = 3.80; 95% CI: 1.43-10.11; p = 0.008). Other income categories were not statistically significant.

**Table 2:** Significance of modifiable factors of individuals linking the likelihood of individuals undergoing genetic testing for cancer. aOR: adjusted odds ratio from survey-weighted multivariable logistic regression; CI: confidence interval. Significance defined as p < 0.05.

Category (reference)	aOR	95% CI	p-value	Significant
≤USD $10,000 (ref)	1.00 (ref)			
USD $10,000–$19,999	0.97	0.37–2.53	0.948	No
USD $20,000–$34,999	3.8	1.43–10.11	0.008	Yes
USD $35,000–$74,999	1.56	0.57–4.24	0.382	No
≥USD $75,000	1.42	0.45–4.44	0.548	No
Trust in healthcare providers (higher vs lower)	0.18	0.04–0.87	0.033	Yes (inverse)
Use of wellness apps (yes vs no)	2.12	1.05–4.26	0.036	Yes
Looked for health info online (yes vs no)	1.35	0.55–3.33	0.51	No
Medical bill difficulty (yes vs no)	1.28	0.59–2.76	0.533	No

Two behavioural factors showed significant associations. Use of health/wellness apps was positively associated with testing (aOR = 2.12; 95% CI: 1.05-4.26; p = 0.036). Conversely, higher trust in healthcare providers was inversely associated with genetic testing uptake (aOR = 0.18; 95% CI: 0.04-0.87; p = 0.033).

**Figure 1:**
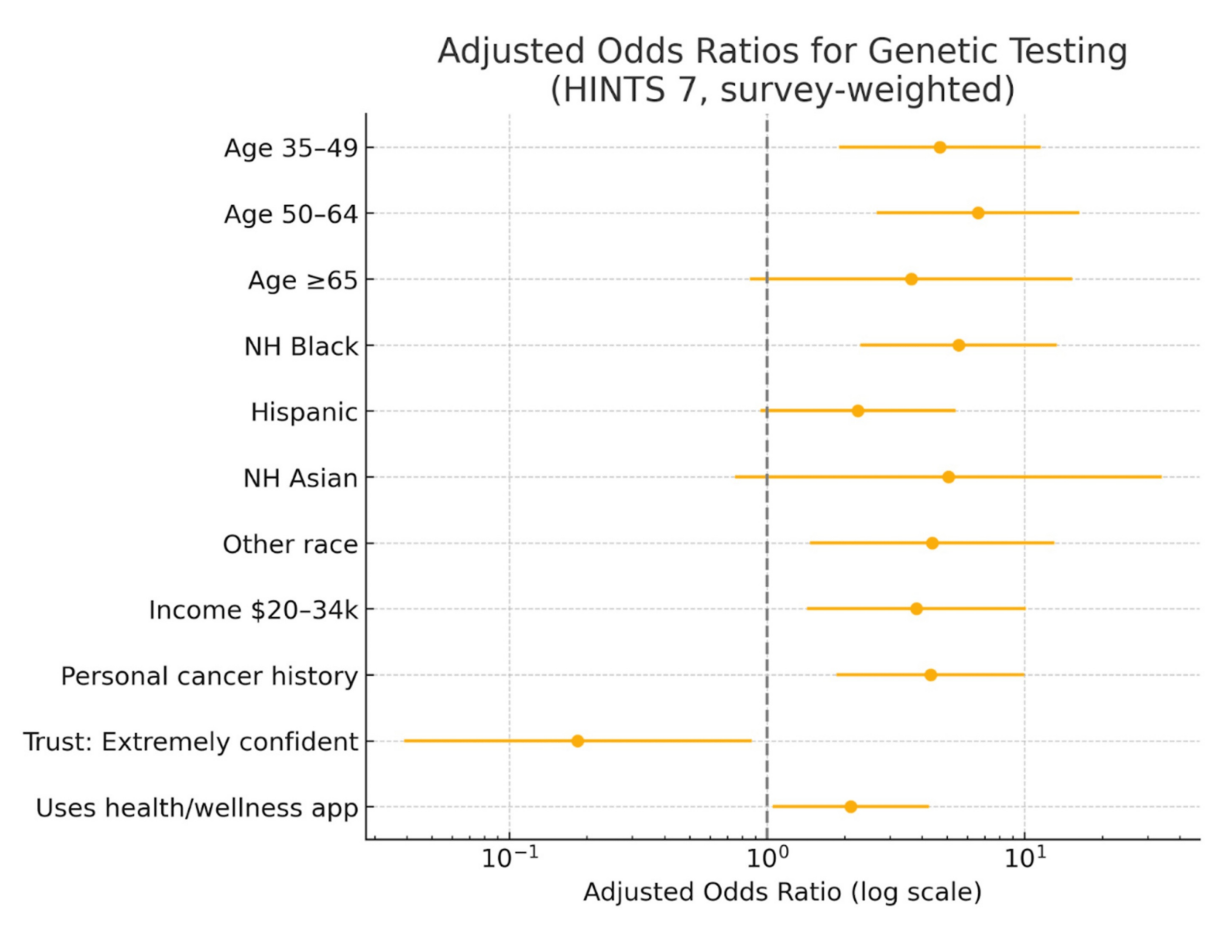
Adjusted odds ratios (log scale) from a survey-weighted multivariable logistic regression of ever having had cancer genetic testing, HINTS 7 (2024). Points show aORs; bars show 95% CIs; vertical line = 1. Reference groups: age 18–34, male, non-Hispanic White, income <$10,000. Trust in providers = “extremely confident” vs others; wellness app use = yes vs no; N = 514 (weighted to U.S. adults). NH: non-Hispanic; HINTS 7: Health Information National Trends Survey 7, aORs: adjusted odds ratios.

The survey-weighted forest plot displays adjusted odds ratios (log scale) and 95% CIs from the multivariable model, with the vertical line marking the null (aOR = 1) (Figure 1). Higher uptake was observed for adults aged 35-49 (aOR = 4.69; 95% CI: 1.90-11.56) and 50-64 (aOR = 6.60; 2.67-16.33), for non-Hispanic Black adults (aOR = 5.55; 2.31-13.35) and those identifying as “Other” race/ethnicity (aOR = 4.37; 1.46-13.06), for incomes $20-34k (aOR = 3.80; 1.43-10.11), for participants with a personal cancer history (aOR = 4.32; 1.86-9.99), and for users of health/wellness apps (aOR = 2.12; 1.05-4.26); CIs for these estimates do not cross 1.

In contrast, “extreme” trust in healthcare providers was inversely associated with testing (aOR = 0.18; 0.04-0.87). Estimates for age ≥65, female sex, Hispanic and non-Hispanic Asian groups, other income categories, family history of cancer, self-rated health, seeking health information online, and medical bill difficulty were not statistically significant, as their CIs include 1.

Key findings

Uptake of cancer genetic testing was significantly higher among middle-aged adults, non-Hispanic Black individuals, those with moderate household incomes, and participants with a personal history of cancer.

Engagement with digital health tools was a positive predictor, while higher trust in healthcare providers unexpectedly correlated with lower testing uptake. These results highlight complex demographic and behavioural influences on testing utilisation, underscoring the need for targeted outreach strategies to ensure equitable access.

## Discussion

This study helps shed light on what influences cancer genetic testing among U.S. adults and brings attention to some of the gaps and challenges people might face in accessing it. Interestingly, we found that non-Hispanic Black individuals were more likely to report getting genetic testing than White individuals. This could reflect more focused outreach efforts or a growing level of awareness about genetic testing within that community [10]. These findings highlight the need to reduce access gaps and promote fairness in cancer prevention and care. Notably, individuals with a personal history of cancer were more inclined to undergo genetic testing, underscoring the important role of clinical advice and risk-based decision-making [11]. It may seem paradoxical at first because those who had "extreme" confidence in their healthcare professionals had decreased probabilities of undergoing genetic testing. One possible explanation is that older adults, who often report high levels of trust in providers, may also have lower health literacy than younger individuals [12]. Some past studies show that people with better health knowledge usually feel more satisfied with their care and trust their doctors more. On the other hand, those with less health literacy might rely too much on their providers and not seek out health services on their own [13]. In our study, we also noticed that people who use health or wellness apps were more likely to get genetic testing for cancer risk. This might mean that those who are more involved in managing their health are also more likely to take steps like getting tested.

The increase in demand for genetic counselling tests and programs has consequently led to the involvement and training of more board-certified genetic counsellors to meet the demand and bridge the gap between the availability and the actual execution of these tests [14]. Introduction of telemedicine has reduced the geographical barriers while accessing these tests by serving those in remote locations [15]. Attempts should be continued to make genomic literature available to the concerned population and their corresponding healthcare providers so that it enables them to make well-informed rounded decisions with regards to precision medicine and genetic testing. Making genetic testing a regular part of routine health checkups, just like how normal blood reports are analysed, financial remuneration to minimise out-of-pocket expenses, and introduction of suitable educational interventions could enhance the rates of testing for anyone who might need it. This development and changing approach between genomic discoveries and precision medicine unfolds an era of customised healthcare suitable to individual preferences and requirements instead of a generalised umbrella treatment plan [16].

Incorporation of genetic screening methods is introduced with the intention of reducing morbidity due to genetic conditions and critical illnesses [17]. However, this can be considerably challenging when it comes to executing it without biases of ethical, social, and economic norms. Ultimately, the true realisation of precision medicine will reach its maximum potential under the concerted efforts of interdisciplinary medical teams that aim to erase the existing disparities, encourage inclusivity believe that all individuals can positively benefit from these transformative reforms in medicine [18,19].

Attention should be paid while collecting data to ensure that diverse datasets are included. This also highlights the need for continuous research and targeted interventions to realise the full potential of precision medicine and improve cancer prevention and treatment options on a grassroot level [20-22]. Challenges related to these determinants need to be prioritised and treated as a global concern [23].

There is a rising trend for the extension of tailored healthcare according to individual needs in medical practice and the governing bodies have allowed genetic and molecular testing methods in pharmaceutical product labels to minimise risks and augment treatment efficacy [24]. The genetic profile of an individual is often exploited in customised medicine to prevent, diagnose, and treat genetic illnesses [25]. Nevertheless, precision medicine can sometimes have ethical concerns and these need to be acknowledged with sensibility [26].

It is imperative that we recognise the limitations of this study. Since HINTS 7 is a cross-sectional dataset, it can highlight associations between predictors and genetic testing outcomes, but it cannot confirm any cause-and-effect relationships. As the study relied on a modest number of people and it included self-reported data, it has potential for selection and recall bias, which can interfere with the overall generalisability of the findings. In future studies, researchers should use longitudinal research methods and improve how they collect data, such as including actual medical records and more objective measurements, to minimise the risk of potential biases. This gap between the demand and necessity for genetic testing and its availability is evident in the racial and ethnic minorities, where such populations remain deprived due to geographical or religious barriers and are faced with challenges while accessing genomic medicine [27]. An estimated 5-10% of cancers that are linked to genetic inheritance, with such patients qualifying to be tested according to the medical genomics guidelines, do not undergo clinical testing, contributing to the existing disparities [28].

## Conclusions

In conclusion, genetic testing for cancer risk among the U.S. adult population is dependent on a number of variables--sociocultural, demographic, and behavioural patterns are to name a few. Individuals with above-average household incomes, health app users, family/personal history of cancer diagnoses, middle-aged adults, and the non-Hispanic Black population are more likely to undergo such testing as a direct correlation to their health awareness. This interestingly displays a striking contrast in that those who place a higher degree of faith in their healthcare providers are less likely to undergo genetic testing suggesting that trust in traditional medical advice can influence the perceived requirement and reduce the numbers in which such individuals who show up for testing. These results highlight the importance of education and accessibility of information surrounding the decisions influencing genetic testing.
